# Two-faced Janus SkQ1/SkQR1: patterns and molecular volume threshold for substrate recognition by the AcrAB-TolC pump

**DOI:** 10.3389/fphar.2025.1480955

**Published:** 2025-03-06

**Authors:** Marina V. Karakozova, Alexandra I. Sorochkina, Pavel A. Nazarov

**Affiliations:** ^1^ Department of Bioenergetics, Belozersky Institute of Physico-Chemical Biology, Lomonosov Moscow State University, Moscow, Russia; ^2^ Laboratory of Genomics and Biochemistry of Medicinal Plants, All-Russian Scientific Research Institute of Medicinal and Aromatic Plants, Moscow, Russia

**Keywords:** efflux pump, AcrAB-TolC, multidrug resistance, SkQ, antibiotic

## Introduction

Recently, it has become increasingly clear that bacterial antibiotic resistance poses a threat to our secure future. The World Health Organization recognized antibiotic resistance as the cause of the crisis in modern medicine and proposed a Global Plan to Combat Antimicrobial Resistance (WHO, 2015). However, it has already become clear that the problem stems not only from the decrease in the number of new antibiotics in the development pipeline (Plackett, 2020; Randall and Davies, 2021) and their limited success in clinical trials (Theuretzbacher et al., 2019), nor from a sudden emergence of antibiotic resistance, but also from irresponsible use of antibiotics and insufficient understanding of bacterial resistance mechanisms (Nazarov, 2018). The contribution to the general mechanism of resistance can be adequately assessed only for particular factors, but not all of them, due to insufficient knowledge of their features.

Antibiotic targets are often located within the bacterial cell envelope, and antibiotics must overcome a complex cell wall to reach their sites of application. At the same time, antibiotics also need to avoid recognition by multidrug resistance pumps, the main purpose of which is protecting the cell from the action of various xenobiotics (Nazarov, 2022). The contribution of these factors to antibiotic accumulation rate can be represented by the following equation:

$$V\left(с\right)=V\left(p\right)-V\left(e\right)$$

where *V(c)* is the rate of antibiotic accumulation inside the cell, *V(p)* is the rate of penetration of the substance through the cell envelope, *V(e)* is the integral speed of efflux of this antibiotic via the MDR pumps. If *V(e)* ≥ *V(p)* the cell is resistant to the action of the antibiotic, regardless of its mechanism of action and presence of the appropriate resistance genes. In this case, *V(p)* depends only on the physicochemical properties of the substance itself and its concentration outside the cell, while *V(e)* depends on several factors: (1) the number of working pumps, (2) the ability of the pump to recognize substances, (3) the energy state of the cell, since efflux is an energy-dependent process. Thus, the ability of bacteria to survive antibiotic therapy is highly dependent on the ability of their pumps to recognize antibacterial agents.

AcrAB−TolC, a major efflux pump in gram-negative bacteria, consists of the inner membrane protein AcrB (113.6 kDa or 1,049 aa), the membrane fusion protein AcrA (42.2 kDa or 397 aa), the membrane fusion protein TolC (53.7 kDa or 493 aa) and pumps out a large variety of substrates (Jang, 2023). The AcrB protein consists of three domains: the transmembrane domain (TMD), the porter domain (PD), and the funnel domain (FD). The TMD is responsible for proton transfer, the PD is responsible for recognizing substrates and binding to them, and the FD is responsible for transferring the substrate molecule to the TolC protein. The PD has 2 substrate-binding pockets: the proximal binding pocket (PBP) and the distal binding pocket (DBP), which are separated by 11 amino acids termed “switch loop.”

Although the process of substance transfer using the AcrAB-TolC pump has been intensively studied and is generally well understood (Pos, 2024), there are still points that are not completely clear at present. Recognition of substances by this pump begins with the binding of the substrate to the AcrB protein; then, through a series of conformational rearrangements of the pump, the substrate is transferred to the TolC protein (Husain and Nikaido, 2010; Husain et al., 2011; Nakashima et al., 2011). This seemingly simple and understandable pipeline has some subtle points that are not entirely obvious. Based on a computational analysis of the structure of the AcrB protein, it was assumed that the protein contains 4 channels (CH1÷4) for binding and transporting substrates. CH1 allows substrates to enter AcrB through the outer leaflet of the inner membrane (Sennhauser et al., 2006). CH2 leads the substrate to pass to the PBP (Seeger et al., 2006; Zwama et al., 2018). CH3 can guide substrates directly to the DBP by passing the PBP. CH4 also allows substrates to pass to DBP (Oswald et al., 2016; Tam et al., 2021). AcrAB–TolC pumps have a wide range of substrates that include many antibiotics and chemical substances, such as chloramphenicol, ampicillin, florfenicol, puromycin, erythromycin, rhodamine 6G, crystal violet, SDS, tetraphenylphosphonium, cyclohexane, indole, SkQ1 and hexane (Sulavik et al., 2001; Pos, 2009; Nazarov et al., 2017; Jang, 2023).

SkQ is a class of mitochondria-targeted antioxidants developed under the leadership of Academician of the Russian Academy of Sciences Vladimir Skulachev, the best known of which is SkQ1 (Skulachev, 2007). SkQ1 consists of a quinone moiety and a phosphonium moiety linked by a 10-mer alkyl linker. At first, the mitochondria-targeted antioxidants and antibiotics, including SkQ1, were believed to have no antibacterial properties (Anisimov et al., 2011), then SkQ1 was assumed to only act against low G+C gram-positive bacteria (Khailova et al., 2015). Further studies showed that SkQ1 acts on both low G+C and high G+C gram-positive bacteria (Nazarov et al., 2023), as well as gram-negative bacteria, with the exception of *Escherichia coli* and related bacteria (Nazarov et al., 2017). It turned out that the key factor is antimicrobial resistance, which arises due to the action of the main MDR pump–AcrAB-TolC (Nazarov et al., 2020).

SkQR1 also belongs to the SkQ class of substances, and has long been considered a fluorescent analogue of SkQ1 (Antonenko et al., 2008; Fetisova et al., 2011). SkQR1 is a derivative of the SkQ1 molecule, whose phosphonium group is replaced with a rhodamine group. Although neither SkQ1, nor SkQR1 were previously believed to have antibacterial properties, the discovery of the antibacterial properties of SkQ1 cast doubt on their supposed absence for its fluorescent analog SkQR1, especially since rhodamine 6G also has an antibacterial effect. As with SkQ1, SkQR1 acts on both high G+C and low G+C gram-positive bacteria (Nazarov et al., 2023; 2024a). In fact, SkQR1 appeared to be identical in properties to its nonfluorescent counterpart, SkQ1.

Although the mechanism of operation of the transporters can be conceived (Pos, 2024), the principles of the substrate recognition still remain a mystery. Substances acting as substrates for the specific pump differ in size, hydrophilicity, charge and chemical groups composition, which makes empirical prediction difficult. Analysis of physicochemical properties of various substrates makes it possible to divide them into groups, but the principles behind this division are still insufficient for explaining the substrate preferences of various membrane channels (Jang, 2023). Thus, our knowledge prompts the idea that the pump recognizes patterns of chemical structures that bind to the channels active site. At the same time, substitutions of chemical groups within the patterns may ultimately not affect their recognition by the pumps. For example, replacing chemical groups in the plastoquinone derivative of the SkQ1 molecule had absolutely no effect on its recognition (Nazarov et al., 2024b).

## Pattern recognition as a concept

The fact that some substances of similar size and properties are not recognized by AcrAB-TolC pumps suggests that the pump recognizes some chemical patterns by which substrates can contact one or more recognition channels (CH1÷4), which is followed by the process of substrate transfer from AcrB to TolC. If the molecule does not have such recognition patterns, then it is not recognized by the pump and is not pumped out by it. However, it is not clear how many patterns the molecule must have for its successful recognition by the pump, and how these patterns should relate to each other. Accordingly, it can be assumed that if a molecule has one or more recognition patterns, then it should be recognized by the AcrAB-TolC pump.

**FIGURE 1:**
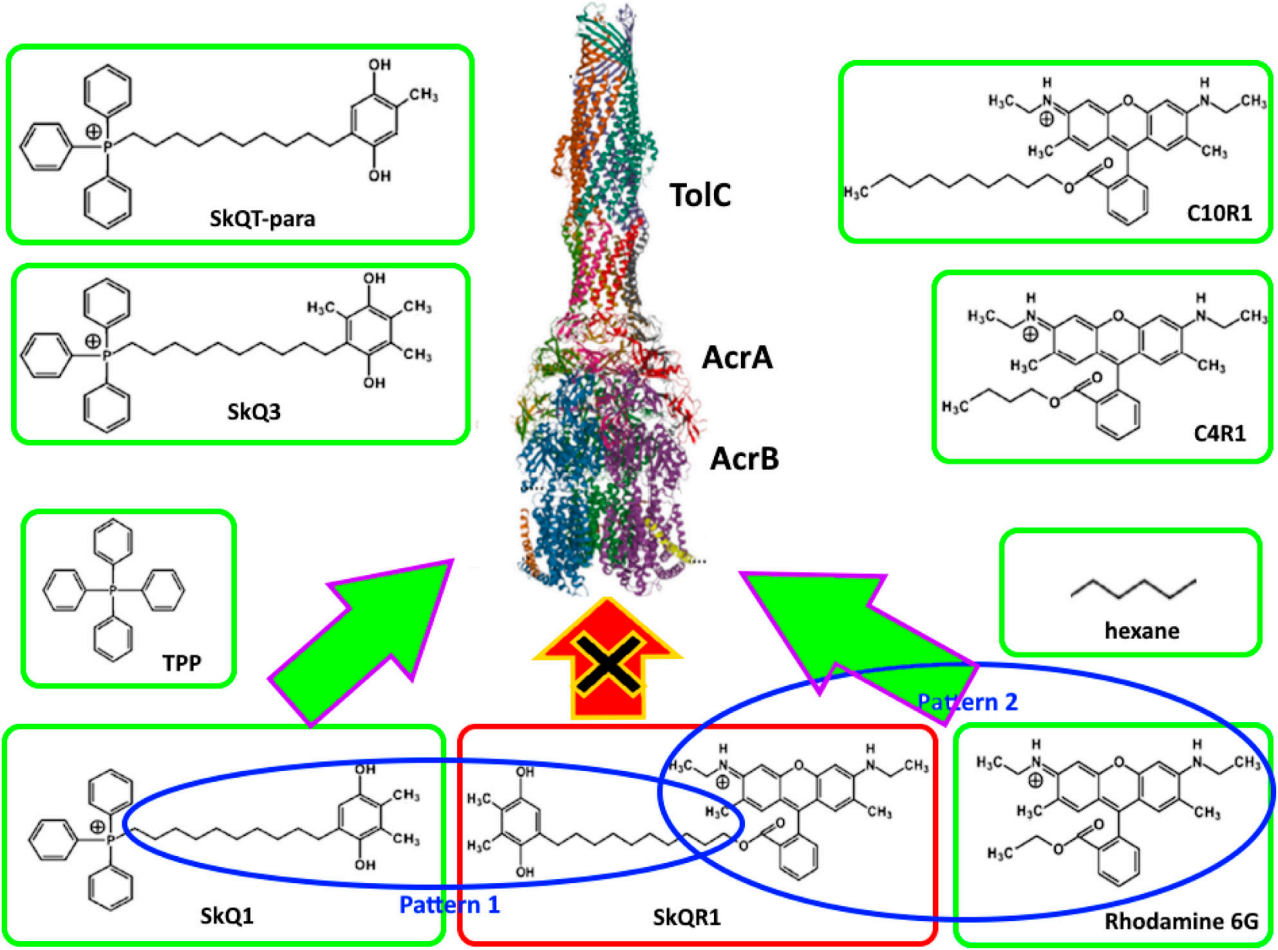
Principles of pattern-based molecular recognition by AcrAB-TolC pump. Green frames indicate AcrAB-TolC pump substrates. Blue ovals indicate similar patterns in SkQR1 and structurally similar substrates. The red frame indicates SkQR1, which is not pumped out despite the presence of the patterns described above.

It can be assumed that SkQ1, being a substrate of the AcrAB-TolC pump, is recognized by one or more substrate recognition channels (CH1÷4). Breaking down the SkQs molecule into patterns (Figure 1) produces a phosphonium pattern, an alkyl linker pattern, and a quinone pattern. According to the logic of pattern recognition, since tetraphenylphosphonium is recognized by the AcrAB-TolC pump, then the triphenylphosphonium part of the SkQs molecule should be recognized by it as well. Similarly, if hexane is recognized by the pump, then the alkyl linker of the SkQs molecule will be recognized by it too. The same logic can be applied to the quinone pattern. Our data on various modifications of the quinone part of SkQ molecules confirms this: since the plastoquinone pattern is recognized by the AcrAB-TolC pump, any modified plastoquinone will be recognized by it too (Nazarov et al., 2024b). However, we would have observed a similar picture even if quinones did not take part in recognition by the AcrAB-TolC pump.

Similar logic could be applied to SkQR1, the fluorescent analogue of SkQ1 (Antonenko et al., 2008). However, existence of more complex principles cannot be ruled out. It is possible that only the end groups of the molecular structure are recognized, and the linker plays a negligible role in recognition. Another possibility could be that one or more of the patterns we have identified may not be recognized, but the substance containing it is still pumped out. This raises the question of the difference between being detected by the pump and being removed by the pump. In this case, all molecules that are pumped out by the pump are recognized by it, but not all molecules with recognizable patterns are expelled out of cells by the pump. So, the general rule would be: if a molecule has a structure that we would consider as recognized by pumps, then this molecule should be removed by pumps. However, is this really happening?

## Rhodamine 6G is recognized by AcrAB-TolC but SkQR1 is not

SkQR1, like SkQ1, has an antibacterial effect against gram-positive bacteria. We could assume the absence of its pronounced antibacterial effect against gram-negative bacteria, same as in the case of SkQ1, which could be explained by the operation of the AcrAB-TolC pump. SkQR1 accumulation in gram-negative cells was shown to be low, as detected with fluorescence correlation spectroscopy, which could also explain its stronger effect on gram-positive bacteria. However, its C4R1 analogue has a similar antibacterial effect against gram-positive bacteria, but accumulates in gram-positive cells worse than SkQR1 in gram-negative ones. Thus, the problem is apparently not due to the process of SkQR1 accumulation in cells, and one could assume that the whole phenomenon is caused by the operation of MDR pumps.

If we split the SkQR1 molecule into patterns (Figure 1), just as we did for SkQ1, we end up with a quinone pattern, an alkyl linker pattern, and a rhodamine pattern. Thus, in SkQ1 and SkQR1, two of the three patterns are identical, and if they are recognized by the AcrAB-TolC pump in SkQ1, then the whole SkQR1 molecule should be recognized by it as well, but this does not appear to happen (Nazarov et al., 2024b). This observation could be explained by the fact that the rhodamine pattern is not recognized by the AcrAB-TolC pump, but this is also not true. Rhodamine 6G is a substrate of the AcrAB-TolC pump (Sulavik et al., 2001), so the rhodamine pattern is also recognized by the AcrAB-TolC pump. It turns out that while all the patterns are recognized by the pump individually, it fails to recognize them together and the molecule consisting of them is not pumped out.

## Discussion

It should be noted that the concept of recognition patterns appears to work effectively. We see predictable results with such molecules as SkQ1, SkQ3, SkQT and SkQThy when the quinone molecule changes slightly. We see the same predictable results in the series of rhodamine derivatives CnR1, where recognition of the compound by the AcrAB-TolC pump decreases with an increase in the alkyl linker length (from C2R1 to C10R1). All of the above undoubtedly suggests that recognition patterns can only play a role within a certain region of the molecule. Thus, there appears to be a certain threshold of molecular volume of the substrate that is recognized by the pump, and above this threshold the substrate becomes unrecognizable. It can be assumed that the threshold of this molecular volume will be determined by the presence of patterns, and it may be different for each.

Despite the great diversity in the structures of pumped substrates, by and large we can only observe the pumping out of toxic substances or fluorescent substrates, whose accumulation in the cell we can detect either directly or through cell death. The pumping out of non-toxic compounds is virtually unnoticeable, but these substances can have a very serious impact on functioning of the pumps, taking up important cellular resources in the form of a proton gradient and ATP. Thus, our general knowledge of pump substrates is a typical example of survivor bias. If the study of rhodamines (Nazarov et al., 2024a) had only considered SkQR1, we would have correctly concluded that SkQR1 is not a substrate of the AcrAB-TolC pump. However, we would have overlooked the fact that the disappearance of recognition by the pump may be due to the size of the molecule and the volume it occupies.

Considering SkQR1 as a fluorescent analogue of the mitochondria-targeted antioxidant and antibiotic SkQ1 appears to be incorrect. In the case of SkQ1, the efflux through the AcrAB-TolC pump was demonstrated directly using ethidium bromide (Nazarov et al., 2017), whereas this could not be shown for SkQR1 due to the lack of difference between the TolC deletion mutant and the wild-type *E. coli*. We observe the accumulation of fluorescent SkQR1 using fluorescence correlation spectroscopy (Nazarov et al., 2024a) in bacterial cells, but again, we do not see a difference between the TolC deletion mutant and the wild-type *E. coli*. The application of the mitochondrial uncoupler carbonyl cyanide m-chlorophenylhydrazone resulted in a decrease in the accumulation of SkQR1, rather than maximal accumulation in the cell (Blair and Piddock, 2016), since the accumulation of SkQs in the cell is an energy-dependent process. Thus, unlike SkQR1, SkQ1 serves as an inhibitor and substrate of the AcrAB-TolC pump. Therefore, considering SkQR1 as a fluorescent analogue of SkQ1 in the case of gram-positive bacteria is possible, while in the case of gram-negative bacteria, it is rather unlikely. Eukaryotic cells differ from prokaryotic cells in the composition of their efflux pumps, so the consideration of SkQR1 as a fluorescent analogue of SkQ1 should be evaluated on a case-by-case basis, as eukaryotic cells vary significantly in the composition and expression levels of pumps even within a single organism.

In this case, information about potential pump substrates becomes very relevant. At the same time, the absence of pumping also gives us the necessary information about spatial restrictions in the operation of pumps. Thus, information that a particular molecule is not a pump substrate is no less valuable than information about its actual substrates. In this case, the most valuable information would be about a variety of substrates that differ slightly from each other, as is the case with SkQs and CnR1. And although we do not yet know the general rules, it cannot be excluded that the volume of molecules plays an important role in pumping out a recognized molecule in one or several recognition channels (CH1÷4). It allows us to understand in more detail the role of patterns and molecular volume in the recognition of substrates and their pumping by MDR pumps.
